# Corrosion of Titanium Electrode Used for Solar Saline Electroflotation

**DOI:** 10.3390/ma16093514

**Published:** 2023-05-03

**Authors:** Felipe M. Galleguillos Madrid, María Arancibia-Bravo, Jonathan Cisterna, Álvaro Soliz, Sebastián Salazar-Avalos, Bastián Guevara, Felipe Sepúlveda, Luis Cáceres

**Affiliations:** 1 Centro de Desarrollo Energético Antofagasta, Universidad de Antofagasta, Antofagasta 1240000, Chile; 2 Departamento de Química, Universidad Católica del Norte, Antofagasta 1249004, Chile; 3 Departamento de Ingeniería en Metalurgia, Universidad de Atacama, Copiapó 1531772, Chile; 4 Departamento de Ingeniería en Minas, Universidad de Antofagasta, Antofagasta 1240000, Chile; 5 Departamento de Ingeniería Química y Procesos de Minerales, Universidad de Antofagasta, Antofagasta 1240000, Chile

**Keywords:** solar electroflotation, titanium electrode, morphological patterns, solar energy, oxyhydrogen gas

## Abstract

The solar electroflotation (EF) processes using saline electrolytes are today one of the great challenges for the development of electrochemical devices, due to the corrosion problems that are generated during the operation by being in permanent contact with Cl^−^ ions. This manuscript discloses the corrosion behavior of titanium electrodes using a superposition model based on mixed potential theory and the evaluation of the superficial performance of the Ti electrodes operated to 4 V/SHE solar electroflotation in contact with a solution of 0.5 M NaCl. Additionally provided is an electrochemical analysis of Ti electrodes regarding HER, ORR, OER, and CER that occur during the solar saline EF process. The non-linear superposition model by mixed potential theory gives electrochemical and corrosion parameters that complement the information published in scientific journals, the corrosion current density and corrosion potential in these conditions is 0.069 A/m^2^ and −7.27 mV, respectively. The formation of TiO_2_ and TiOCl on the anode electrode was visualized, resulting in a reduction of its weight loss of the anode electrode.

## 1. Introduction

Nowadays, as a result of climate change, conventional industrial processes are migrating to greener or carbon-neutral processes [1], to reduce greenhouse gases and the dependence of mining and industrial processes on fossil fuels [2]. Electrochemical processes are increasingly considered in mineral processing [3] because they are environment-friendly and because they have the possibility of being electrically powered by renewable energy [4], mainly solar energy by photovoltaic panels [5]. One of the most widely used electrochemical processes is electroflotation in the industry [6,7].

The electroflotation is an electrochemical process that is based on the principle of electrolysis [8], where the cathodic subprocess generated H_2_ bubbles and the anodic subprocess guarantees the evolution of O_2_ or Cl_2_ bubbles [9,10] depending on Cl^−^ concentration present in the electrolyte. The mixture of these gases generated during solar electroflotation is known as Oxyhydrogen (HHO) [11]. These gases emerge simultaneously from the electrode surface as electrolytic bubbles [12]. The electroflotation process has been applied in (i) solid-liquid of colloidal particles [13,14,15,16] separation, (ii) microalgae [17], (iii) oil [18,19], (iv) mineral recovery [15,20,21,22], (v) microplastic [23,24], and (vi) wastewater [25,26,27,28] among others [29,30].

The solar electroflotation process is a new application and it greatly helps to decarbonize mining and industrial processes [31,32,33]. The use of seawater or brackish solutions is increasingly common [27,34] and this has generated corrosion problems in several processes [35]. Currently, several mineral processes use direct seawater [36,37,38,39,40], therefore, the study of electrode behavior during the solar electroflotation process is very important for to enhancement of the current electrochemical devices.

The Ti materials have been used widely in an extensive diversity of applications product of their mechanical and electrochemical properties. Titanium is stable and generates a protective passive oxide film at the high potential in contact with natural environments including saline solutions such as seawater, geothermal water, or altitude brine [41]. In most natural aqueous environments, the Titanium Oxide is typically found to be TiO_2_ (rutile) but may contain combinations of other titanium oxides as well as TiO, Ti_2_O_3_, and TiO_2_ during the cathodic subprocess and according to the Pourbaix Diagram of Ti in contact with H_2_O and NaCl [42,43]. Lower temperatures often generate more amorphous forms of TiO_2_ (anatine), or a mixture of them (two different crystal structures of TiO_2_) [44]. Much of the information published in popular science journals do not provide detailed and useful information regarding problems and behavior of the materials used as electrodes against corrosion using a non-linear model for calculating the electrochemical and corrosion parameters. The use of seawater exacerbates the corrosion rate of the electrodes [26,45], especially the anodic electrode due to the dissolution of the applied high-potential product material [46]. Normally, the applied potentials vary from 10 to 15 V, however, operating at medium potentials helps to reduce the mass demands of the electrodes. The contribution of the study aimed to investigate the effects of the corrosion behavior of titanium electrodes subjected to a solar direct electroflotation process in contact with an artificial seawater electrolyte using a superposition model according to mixed potential theory and a superficial analysis by SEM and EDS images after solar direct electroflotation process at 4 V/SHE during 1 h of operation.

## 2. Materials and Methods

### 2.1. Corrosion of Ti Rod Electrode Study

The experimental corrosion procedure was designed to examine the kinetic of the partial electrochemical reactions on the Ti alloy electrode immersed in 0.5 M NaCl solutions, focusing the attention on the HER, ORR, and titanium oxidation reaction (TOR). For this purpose, a series of polarization curves were measured in a freshly prepared electrolyte using a Ti rotating disc electrode. The NaCl solution used in all tests was prepared from the analytical grade of NaCl salt (99.9% pure Sigma-Aldrich, Darmstadt, Germany) in deionized water. The Ti alloy electrodes were fabricated from a cylindrical rod with a 4 mm of diameter and a length of 10 mm. This Ti rod from Balance World Inc., USA., was concentrically inserted using resin adhesive in a PTFE tube of 8 mm in diameter that serves as a fixing device to the shaft of the rotating disc electrode cell stands at 20 °C. This temperature was retained using a water-jacketed cell with circulating water through a thermoelectric temperature control device. All the experiments were repeated in triplicate to verify their reproducibility. All potentials reported are referred to as the standard hydrogen electrode (SHE). The electrochemical analysis was performed using a BASI/RDE-2 rotating electrode interphase connected to an Epsilon potentiostat/galvanostat. The electrochemical behavior of Ti was studied by linear sweep voltammetry in a conventional 3-electrode cell system using Ti as a working electrode (WE), Pt wire as a counter electrode (CE), and Ag/AgCl (4 M KCl Sat.) as a reference electrode (RE). The experimental protocol for polarization data was according to previous work [47] in a potential range between −1200 to 200 mV/SHE. Previous to each run the Ti alloy electrode was maintained at −1200 mV/SHE for 30 s. 

### 2.2. Solar Electroflotation Simple Cell

The electroflotation system is composed of two electrodes, which were manufactured by a Ti mesh of 2 cm^2^, and both were immersed in 0.5 M NaCl solution at 20 °C for 1 h. The reactor used has a volume of 100 mL, where the electrodes are located at the bottom of the reactor with a separation of 1 cm between them without a membrane. Both electrodes were connected to a photovoltaic solar system with an output potential of 4 V. The NaCl solution used in all tests was prepared from an analytical grade of NaCl salt (99.9% pure Sigma-Aldrich) in deionized water. All experiments were carried out using room temperature for 1 h each of them, where the cell was opened to the air atmosphere.

### 2.3. Electrodes Surface Characterization

The morphological characterization was studied by scanning electron microscopy (SEM) using a Zeiss EVO MA 10 scanning electron microscope (Zeiss, Oberkochen, Germany) equipped with an energy-dispersive X-ray (EDS) analyzer. 

### 2.4. Weigh-Loss Measurements

**Figure 1:**
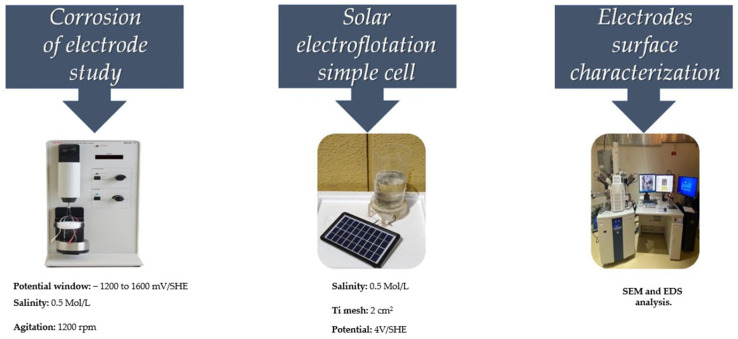
Schematic graph for the experimental preparation for solar EF process.

The weight-loss measurement was carried out by weight loss measurement, where the electrode was weighed before and after each batch process. The expression used to describe the weight loss is defined as follows:

(1) $$W_{loss}=\frac{k\cdot \left(m_{Ti}^{i}-m_{Ti}^{f}\right)}{A\cdot t\cdot \rho }$$

where *k* is a constant of $8.76\cdot 10^{-4}$, $m_{Ti}^{i}$ and $m_{Ti}^{f}$ are the initial and final mass of the electrode in [g], *A* is the area in [cm^2^], *t* is time in [h] and ρ is the density of material in [g/cm^3^], respectively [48] In Equation (1), it is supposed that the surface area of Ti in contact with the saline electrolyte remains constant during the corrosion process, but this is not the situation, as the surface area of Ti in contact with the saline electrolyte will changes as a function of time as the entity dissolves within the corrosive medium. The schematic graph for the experimental preparation of the samples will help to clarify the procedures of the EF process as shown in Figure 1.

## 3. Results and Discussion

### 3.1. Surface and Elemental Analysis after the Corrosion Process

**Figure 2:**
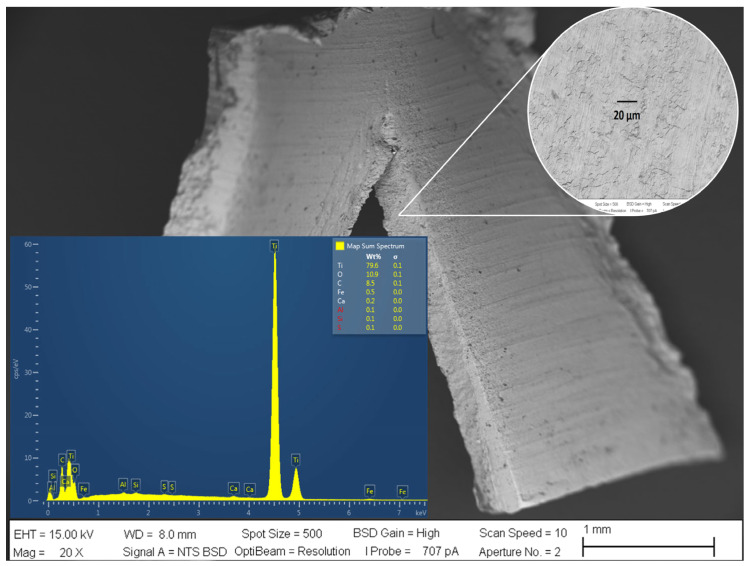
Surface characterization by SEM and EDS of the electrode before solar electroflotation.

Figure 2 shows the material electrodes used for solar direct seawater EF were Ti mesh electrodes from Balance World Inc. 79.6% Ti, 10.9% O, and 8.5% C. The other elements such as Fe, Ca, Al, Si, and S are considered impurities of the Ti electrode.

This section intended to obtain information about a morphological surface change of Ti after the cathodic and anodic subprocesses in contact with a 0.5 M NaCl solution operated at 4 V/SHE [49]. The electrochemical and corrosion parameters from the behaviors of Ti are analyzed and compared with information published in scientific journals. It is well-known that the noble metal Pt is considered a conventional electrocatalyst for HER [50] due to its lower kinetic energy barrier for the dissociation of a water molecule, which is 0.89 eV with the Pt (111) surface [51,52]. The use of noble metals such as Pt reduces the opportunity to industrialize disruptive technologies due to their high cost. The use of Ti as an electrode helps reduce the manufacturing costs of this type of electrochemical device [53] but is a propensity to suffer crevice corrosion and stress corrosion cracking (SCC) [54,55]. The crevice corrosion is divided into three steps: Initiation, propagation, and re-passivation. Before initiation, an aerated saline solution is present on both crevice sides. When passivity is locally broken and the O_2_ that may re-passivate the system is depleted, metal dissolution takes place with the crevice, supported by O_2_ reduction occurring on the surface external to the crevice. At the same time, Ti ions hydrolyze, resulting in acidification of the crevice interior and the deposition of corrosion products once neutral condition. In the propagation step, the ORR occurs at the active site around the crevice. With time, corrosion penetrates the crevice cavity, the ORR decreases, but the HER increases and H_2_ appears. For a longer time, the corrosion is controlled by the ohmic drop between cathodic and anodic causes. After this time, while corrosion can propagate laterally, the maximum penetration depth does not increase anymore. Once the crevice is initiated, propagation is fast, being supported both within the crevice by HER is close to 80% and outside the crevice by ORR is around 30%. The content of Fe impurities in Ti is reported to have a great effect on crevice corrosion. These particles behave as catalytic sites for HER, then increasing the local pH at the propagating sites, reducing corrosion rate and eventually promoting the re-passivation of Ti alloy, according to the following reactions:

(2) $${Ti}^{4+}+{2H}_{2}O\to {\left[{Ti(OH)}_{2}\right]}^{2+}+{2H}^{+}$$

(3) $${Ti}^{4+}+{4H}_{2}O\to {Ti(OH)}_{4}+{4H}^{+}$$

At the same time, the Ti alloy material is generally resistant to stress corrosion cracking (SCC), but under certain conditions appears potential problem. Two mechanisms are considered as the principal damage of material (i) anodic dissolution, and (ii) H_2_ embrittlement. The sources of H_2_ in Ti are multiple: crevice corrosion, once initiated by H^+^ reduction inside the crevice by H^+^ absorption to produce hydride as TiH product according to:

(4) $$Ti+{4H}^{+}\to {Ti}^{4+}+{2H}_{2}$$

The passive Ti layer is promoted by reaction with a neutral medium and proceeds at an extremely slow rate in the range of the passive current density, according to the following reaction.

(5) $$Ti+{2H}_{2}O\to {TiO}_{2}+{2H}_{2}$$

In the first step, the H_2_ is diffused by the TiO_2_ film and then must be absorbed in the Ti-film interface. For absorption to proceed, the reduction of Ti^4+^ to Ti^3+^ at negative potentials. Equation (5) is the global reaction for HER, but it does not occur directly. The mechanisms for H_2_ production are shown in Equations (6)–(9), respectively [56,57,58,59].

(6) $$4H_{2}O+4e^{-}\to 2H_{2}+4{OH}^{-}$$

(7) $$H_{2}O_{\left(l\right)}+M+e^{-}\to {MH}_{ads}+{OH}^{-}$$

(8) $${MH}_{ads}+H_{2}O_{\left(l\right)}+e^{-}↔M+{OH}^{-}+H_{2(g)}$$

(9) $$2{MH}_{ads}↔2M+H_{2(g)}$$

The reactions that occur in the cathodic sub-process are related to the ORR and HER, respectively. One of the primary obstacles in the technology of saline water splitting is the search for cost-effective and effective catalyst materials for ORR. ORR can occur through multiple pathways, such as direct (4e^−^) or indirect (2e^−^) pathways in neutral media, leading to the production of OH^−^ ions. Equation (10) expresses the most energy-efficient reaction during the ORR, due to 4e^−^ intervening during the reaction, not generating parallel reactions [60,61,62].

(10) $$O_{2}+{2H}_{2}O+4e^{-}\to 4{OH}^{-}$$

Equations (11) and (12) show the formation of hydroperoxide $\left(HO_{2}^{-}\right)$, due to intermediates pathways generated during ORR. The formation of $HO_{2}^{-}$ is considered an inefficient pathway due to reduced inefficiently the ddissolvedO_2_ present in the electrolyte [63].

(11) $$O_{2}+{2H}_{2}O+2e^{-}\to H{O_{2}}^{-}+{OH}^{-}$$

(12) $$H{O_{2}}^{-}+{2H}_{2}O+2e^{-}\to {3OH}^{-}$$

Both the direct 4e^−^ and the sequential (2+2) e^−^ pathways have been shown to occur simultaneously over the Ti alloy surface of the cathode electrode. The decomposition of $HO_{2}^{-}$ occurs by following a catalytic reaction.

(13) $$HO_{2}^{-}\to \frac{1}{2}O_{2}+{OH}^{-}$$

**Figure 3:**
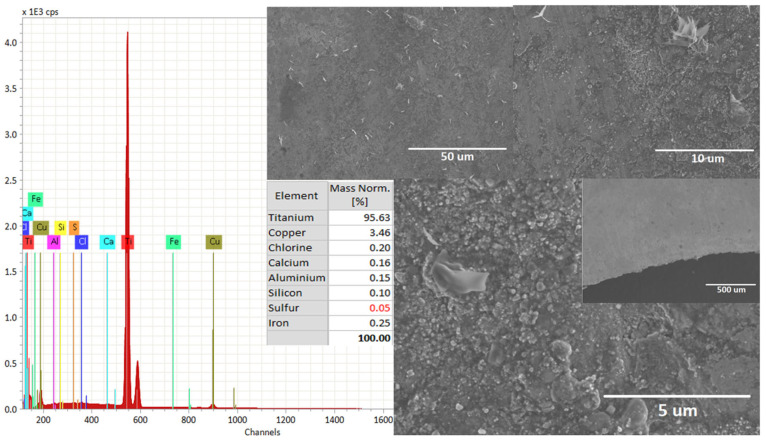
Surface morphology of the cathodic electrode after solar electroflotation process at 4 V and 0.5 M NaCl solution.

The $HO_{2}^{-}the$ formation depends on the Ti alloy (i) surface state, (ii) purity, and (iii) temperature of the electrolyte. Figure 3 shows the SEM images for the cathode electrode material after the solar electroflotation process in 0.5 M NaCl solution for 1 h, and 4 V/SHE applied. In the figure, it is possible to see the morphological surface changes product of the mix of the mechanism of crevice corrosion and SCC over the electrode surface. The surface morphology of the Ti alloy used as a cathode electrode remained largely unchanged from its initial surface conditions.

**Figure 4:**
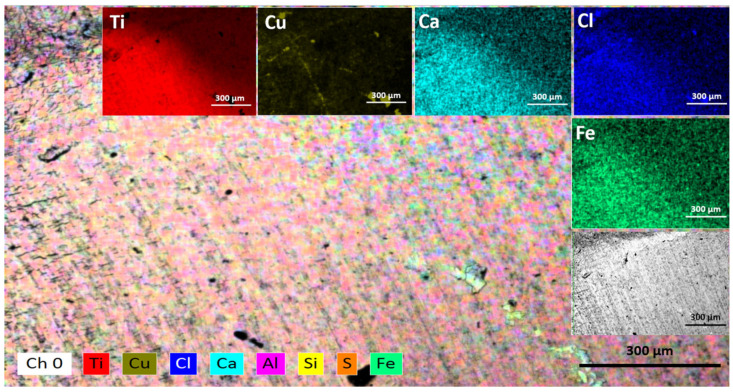
EDS elemental mapping for Ti alloy electrode after cathodic subprocess in the solar electroflotation process at 4 V and 0.5 M NaCl solution. (Ti: line red, Cu: line brown, Ca: line calypso, Cl: line blue, and Fe: line green).

The EDS mapping shown in Figure 4, indicates the elemental analysis and modifications of the Ti alloy surface after the solar electroflotation process. The elements as Fe, Ca, Al, Si, and S are impurities, which can come from air pollution in the local city. Furthermore, the HER and ORR are simultaneous mechanisms reactions as a result of the release of H_2_ microbubbles from the surface of the cathode electrode. It is necessary more study the effect of cationic ions as mono-, di-, and multi-valent ions over the surface of the cathode electrode for efficient HER in seawater.

Additionally, from Figure 2, it is possible to compare changes concerning Figure 3 on the metallic surface of the electrode, which can be caused by the HER and ORR reactions mechanisms on the electrode, generating a volume of gas equal to 60 mL H_2_/mol in 1 h of electrolysis operation calculated by Faraday law and the reduction of pure Ti is according to 95.63% of Ti after cathodic sub-process. The content of Fe in Ti improves the performance of HER product at the catalytic site on the cathode electrode during the solar electroflotation process at 4 V/SHE.

The anodic sub-process is a result of multiple reactions involving charge transfer products that affect the surface of the Ti anode electrode, leading to weight loss and the formation of Ti complex solid products by various mechanisms. Equation (14) represents the overall reaction for OER, while Equations (15)–(19) define the reaction steps that occur during the release of O_2_ bubbles from the anode electrode surface.

(14) $$4{OH}^{-}\to 2O_{2}+2H_{2}O+4e^{-}$$

(15) $$Ti+{OH}^{-}\to {Ti-OH}_{ads}+e^{-}$$

(16) $${Ti-OH}_{ads}+{OH}^{-}\to {Ti-O}_{ads}+H_{2}O_{\left(l\right)}{+e}^{-}$$

(17) $$2{Ti-O}_{ads}\to 2Ti+O_{2(g)}$$

(18) $$Ti-O+{OH}^{-}\to Ti-OOH+e^{-}$$

(19) $$Ti-OOH+{OH}^{-}\to Ti+O_{2(g)}+H_{2}O_{\left(l\right)}+e^{-}$$

where Ti-OH, Ti-O, and Ti-OOH are intermediate products. In parallel occur other important reactions over the surface electrode, such as (i) the dissolution reaction of Ti alloy generating reactive ions according to Equation (20), at the same time, (ii) the dissolution reaction process entails the formation of Ti(OH)_n_ with the subsequent reactions between reactive ions, such as Ti^2+^, Ti^3+^, and Ti^4+^ and the OH^−^ ions generate during the cathodic sub-process. The dissolution anode mechanism expressed in Equation (20) is controlled by change transfer during the solar electroflotation process formed by an electro coagulant, (iii) the CER and hypochlorite production, according to Equations (22) and (23) [35], (iii) TiO_2_ formation over surface electrode according to Equation (24) [64], and (iv) TiO_2_Cl formation over TiO_2_ surface according to Equation (25) [65].

(20) $$Ti\to {Ti}^{n+}+{ne}^{-}\;(n:2,3,and\;4)$$

(21) $${Ti}^{n+}+n{OH}^{-}\to Ti{\left(OH\right)}_{n}+ne^{-}\;;(n:2,3,and\;4)$$

**Figure 5:**
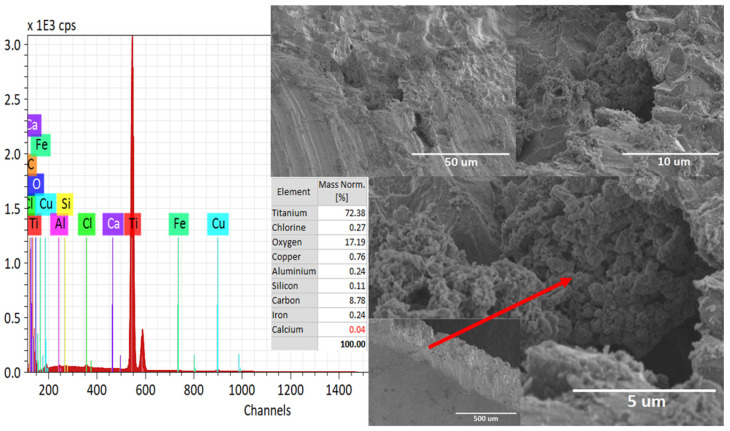
Surface morphology of the anodic electrode after solar electroflotation process at 4 V/SHE and 0.5 M NaCl solution.

**Figure 6:**
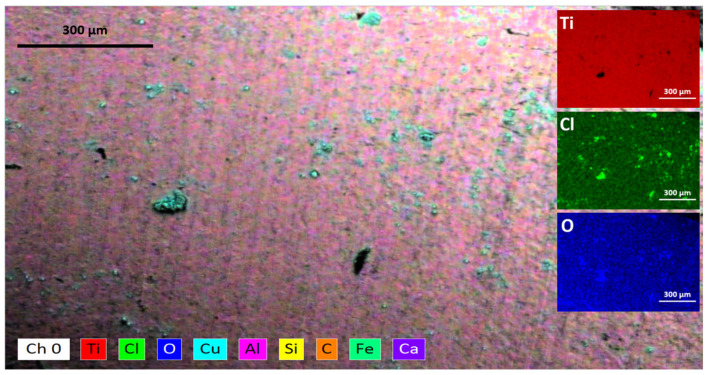
EDS elemental mapping for Ti alloy electrode after anodic subprocess in the solar electroflotation process at 4 V/SHE and 0.5 M NaCl solution. (Ti: line red, O: line blue, and Cl: line green).

The density of microbubbles of O_2_ released from the Ti alloy electrodes during the experiment continuously depends on the applied potential, but the formation of various Ti products over the anode surface can alter the behavior of the electrode during the OER process. The dissolution process is likely due to the transfer of charge or applied potential and the attack of Cl^−^ ions. The application of anodic potential on the Ti alloy electrode results in pitting corrosion and cracks, as shown in Figure 5. As a batch process, the concentration of Cl^−^ ions in the electrolyte decreases over time due to the oxidation of Cl^−^ ions to Cl_2_ gas. Another way is the reduction of Cl^−^ ions to ${OCl}_{\left(aq\right)}^{-}$ formation in the Ti alloy electrode interface [66]. Figure 6 shows the EDS element mapping which indicates the formation of such ${TiO}_{2-x}$ and ${Ti}_{x}O_{Y}Cl$ [65] as a passive film over an anode electrode surface according to the following set of reactions [67,68].

(22) $${Cl}_{\left(aq\right)}^{-}+{OH}_{\left(aq\right)}^{-}\to {OCl}_{\left(aq\right)}^{-}+H_{2}O_{\left(l\right)}+2e^{-}$$

(23) $${2Cl}^{-}+{2e}^{-}↔{Cl}_{2}$$

(24) $$Ti+(2-x)H_{2}O\to TiO_{2-x}+2(2-x)H^{+}+(2-x)e^{-}$$

(25) $$TiO_{2}+{Cl}^{-}\to TiO_{2}Cl+{2e}^{-}$$

According to the EDS mapping expressed in Figure 6, it is possible to suppose the presence of ${TiO}_{2-x}$ and ${Ti}_{x}O_{Y}Cl$ solid products according to the reactions expressed in Equation (24) and to a lesser degree the formation of complex products based on Ti and Cl^−^ according to Equation (25). The above can be related to Figure 5, because it is possible to visualize malformations resulting from the TOR, CER, and OER mechanisms occurring simultaneously on the anode electrode, generating an average weight loss of about $1.05\cdot 10^{-7}\left[mm/y\right]$ in 1 h of solar electroflotation operation at 4 V/SHE in 0.5 M NaCl, respectively.

The reduction in the concentration of Cl^−^ ions is due to the transformation into OCl^−^, Cl_2,_ and to a greater extent, the formation of TiO_2_Cl over the surface of the TiO_2_, which was generated during the TOR mechanism. 

### 3.2. Electrochemical Measurements 

A kinetic study was accomplished by applying non-linear fitting to experimental polarization data, considering the superposition model and mixed potential theory according to the methodology described in our previous works in terms of charge transfer, mass diffusion, and passivation mechanism controls [47,69,70,71,72]. Applying the non-linear fitting methodology, a set of kinetics expressions for the cathodic ORR, HER, and TOR, in terms of the current density was expressed as follows:

(26) $$i=i_{O_{2}}+i_{H_{2}}+i_{Ti}$$

where i is the total current density, $i_{O_{2}}$ and $i_{H_{2}}$ are the partial reduction current densities for ORR and HER, respectively, and $i_{Ti}$ is the partial oxidation current density for the Ti. Although values for $i_{O_{2}}$, $i_{H_{2}}$, and $i_{Ti}$ cannot be experimentally measured, they can be inferred considering kinetics expressions for each one of them. While the partial reactions for HER and TOR follow a charge transfer kinetic mechanism, the case ORR which is significantly affected by O_2_ mass transfer restrictions in the liquid phase requires a kinetic expression for a mixed mechanism of charge transfer and diffusion control. The expressions for each partial reaction are [71]:

(27) $$i_{H_{2}}=i_{0,H_{2}}exp\left(-\frac{2.303\cdot \eta_{H_{2}}}{t_{H_{2}}}\right)$$

(28) $$i_{O_{2}}=\frac{i_{0,O_{2}}exp\left(-\frac{2.303\cdot \eta_{O_{2}}}{t_{O_{2}}}\right)}{1+\frac{i_{0,O_{2}}exp\left(-\frac{2.303\cdot \eta_{O_{2}}}{t_{O_{2}}}\right)}{i_{l,O_{2}}}}$$

(29) $$i_{Ti}=i_{0,Ti}exp\left(\frac{2.303\cdot \eta_{Ti}}{t_{Ti}}\right)$$

where, $i_{0{,O}_{2}}$, $i_{0,H_{2}}$, $i_{0,Ti}$ are the exchange current densities for ORR, HER, and TOR respectively, $i_{l,O_{2}}$ is the limit current density for ORR, $\eta_{O_{2}}=E-E_{eqO_{2}}$, $\eta_{H_{2}}=E-E_{eqH_{2}}$ and $\eta_{Ti}=E-E_{eqTi}$ are the ORR, HER, and TOR overpotentials, respectively. The $E_{eqO_{2}}$, $E_{eqH_{2}}$, $E_{eqTi}$ are the equilibrium potentials for ORR, HER, and TOR, respectively. The $t_{O_{2}}$, $t_{H_{2}}$ are the cathodic Tafel slopes for ORR and HER respectively, and $t_{Ti}$ is the anodic Tafel slope for the TOR. The $E_{eqO_{2}}$, $E_{eqH_{2}}$, $E_{eqTi}$ for ORR, HER, and TOR are, −368 mV, −1804 mV, and 863 mV (Ti^2+^ + 2e^−^ → Ti), respectively.

**Figure 7:**
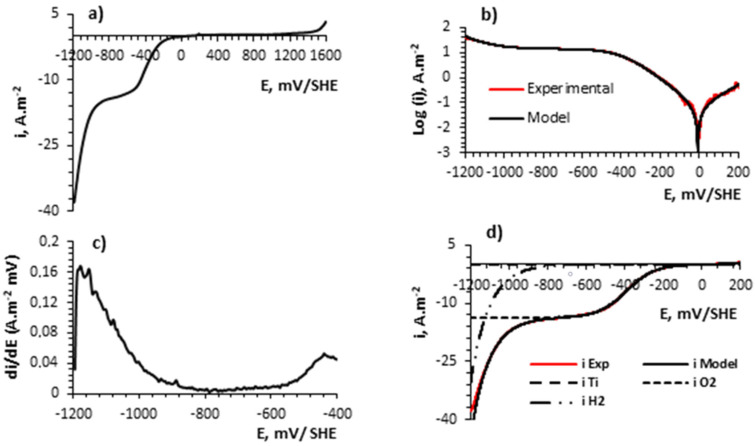
(**a**) Linear polarization curve, (**b**) Tafel polarization curve, (**c**) Slope—Potential curve, and (**d**) superposition model output curves for Ti alloy electrode in aerated 0.5 M NaCl and 2 mV/s.

Figure 7 provides important information about the electrochemical–kinetic behavior of Ti when used as both cathode and anode electrodes in contact with a 0.5 M NaCl solution during solar electroflotation. Figure 7a shows the linear sweep voltammetry (LSV) where the potential window applied in the experiments was between −1200 to 1600 mV/SHE. During ORR [62], the $i_{l}$ is −13.68 A m^−2^. The resistance of the Ti alloy electrode in contact with the NaCl solution reveals values of $E_{corr}$ and $i_{corr}$ equals to −7.27 mV/SHE and 0.069 A m^−2^, respectively. The $i_{corr}$ value is similar (0.068 A m^−2^) reported using Ti-Ni alloy electrodes in contact with 0.5 M NaCl solution [73]. These low and unchanged $i_{corr}$ and $E_{corr}$ values indicate a low corrosion rate, which is supported by the performance of the Tafel curve shown in Figure 7b and the electrochemical kinetic parameters for TOR, HER, and ORR tabulated in Table 1. Figure 7c shows the performance of di/dE (A m^−2^ mV^−1^) for various potentials from cathodic to anodic direction. Three regions of interest are identified: (i) in the potential range between −1200 to −1000 mV/SHE, a single electron is transferred for •OH formation; (ii) between −1000 to −550 mV/SHE, 2e^−^ are transferred for the formation of H_2_O_2_ and HO_2_^−^ (Equations (10) and (11)), which favors the generation of oxidizing products, leading to subsequent corrosion of the surface of the cathode material in contact with the saline solution; and (iii) between −550 to −1600 mV/SHE, 4e^−^ are transferred for the OER reaction [74]. Figure 7d shows the superposition model output curves for the Ti alloy electrode in aerated 0.5 M NaCl at 2 mV/s and the performance of partial current density of H_2_, O_2_, and Ti alloy, respectively. The experimental data match well with the fitted data.

Table 1 shows the principal corrosion and kinetic parameters recollected from the superposition model based on Equations (26)–(29) in terms of charge transfer, mass diffusion, and dissolution mechanism controls.

The values from Table 1, depend on multiple factors such as (i) superficial treatment of the electrode, (ii) temperature of the electrolyte, and (iii) pH of the electrolyte can contribute to changing the electrochemical and corrosion parameters to compare with published information.

Abdellatif El-Ghenymy et al. [35] published a manuscript related to the corrosion behavior of pure titanium anodes in a saline medium that is complementary to the current study. There are many studies regarding electrocoagulation using various materials as electrodes, such as pure Al and Fe. The information regarding the superficial changes of the Ti electrodes due to contact with Cl^−^ ions present in the saline solution and the 4 V/SHE applied directly from the panels solar is considered an important contribution to the development of the EF process. The current work demonstrated that applying a mixed potential model the effects of salinity on the surface of titanium materials used as household appliances, under conditions of ionic strength similar to seawater. Applying a mixed potential theory, as a novel way of evaluating the effects of Cl^−^ ions on corrosion, makes it possible to provide a real contribution to the understanding of the behavior of this type of material subjected to direct current without going through a rectifier, in turn, giving, the versatility of being able to use renewable energy for its operation.

Although no elemental analysis is carried out on the influence of the Cl^−^ ion over time, Figure 5 and Figure 6 can be considered valuable information, since the deterioration of the surface of the anodic material is due to the action of the Cl^−^ ion and the potential applied during the EF process, taking this as a first approximation to the real effect of the Cl^−^ ion. The SEM and EDS images shown in Figure 5 and Figure 6 exhibits Cl deposition over the anode surface in the form of TiOCl and its derivatives. In future studies we will further investigate the effect of the Cl^−^ ion on the deterioration of the anode electrode, however, this material operated for more than 1h at working potential without excessive weight loss of the electrode, being the most important means for study the Ti material in this aggressive condition.

## 4. Conclusions

Direct solar electroflotation is a promising alternative for the production of green H_2_, utilizing solar energy as the primary source. This technology can be a technically and economically feasible option for mineral and industrial processes located in areas with high solar radiation, such as the Atacama Desert in northern Chile. The use of brines or saline solutions for electrolysis presents comparative advantages over pure or freshwater, particularly in mineral processing. The Ti anode electrode is corrosion-resistant when operated at 4 V/SHE using a direct photovoltaic system and artificial seawater, resulting in minimal weight loss considering the most important means for the development of the EF technology. The application of a non-linear model based on mixed potential theory is a real contribution due to the less information about the corrosion performance of electrodes in the EF process. This electrochemical technology can significantly reduce the environmental impact compared to conventional flotation processes, particularly in terms of electrical energy consumption. Additionally, the HHO generated can collaborate with the decarbonization of the mining industry used as solar fuel for in-situ H_2_ recovery. Overall, this study highlights the potential of solar electroflotation as a sustainable and environmentally friendly alternative in the production of H_2_ for various industrial applications.

## Figures and Tables

**Table 1:** Fitted electrochemical and corrosion parameters for Ti mesh electrode in 0.5 M NaCl.

Electrochemical Parameters							
Ti	$i_{{0,T}_{i}}$	$t_{T_{i}}$	$i_{{0,O}_{2}}$	$t_{O_{2}}$	$i_{{1,O}_{2}}$	$i_{{0,H}_{2}}$	$t_{H_{2}}$
	(Am^−2^)	(mVdec^−1^)	(Am^−2^)	(mVdec^−1^)	(Am^−2^)	(Am^−2^)	(mVdec^−1^)
	1.99 × 10^−9^	238	−4.42 × 10^−7^	−168	−13.68	−4.5 × 10^−7^	−217
Corrosion Parameters							
Ti	$E_{corr}$	$i_{corr}$					
	(mV/SHE)	(Am^−2^)					
	−7.27	0.069					

## Data Availability

Data sharing is not applicable.
